# Unveiling the Role of Vitamin D/VDR in Promoting Endometrial Decidualization

**DOI:** 10.1155/ije/1712178

**Published:** 2026-02-23

**Authors:** Jing Guo, Xiangming Tian, Hailong Liu, Qun Lu, Guangming Cao

**Affiliations:** ^1^ Center for Reproductive Medicine, Beijing Chao-Yang Hospital, Capital Medical University, Beijing, China, ccmu.edu.cn; ^2^ The Department of Surgery, Faculty of Medicine, The University of Hong Kong, Hong Kong, China, hku.hk

**Keywords:** decidualization, endometrial stromal cells, infertility, vitamin D, vitamin D receptor

## Abstract

Vitamin D’s impact on reproductive health, particularly endometrial receptivity, has attracted significant attention. This study investigated the effects of vitamin D and its receptor (VDR) on decidualization in human endometrial stromal cells (HESCs). An in vitro decidualization model was established by culturing immortalized T‐HESC or primary HESC in differentiation medium, treated with different concentrations of 1,25(OH)_2_D. VDR expression was modulated using siRNA, and cell morphology was analyzed by immunofluorescence. Decidualization markers (PRL and IGFBP1), vitamin D metabolic enzymes (CYP27B1 and CYP24A1), and VDR were measured using Western blot, qPCR, and ELISA. Aromatase (CYP19), estrogen receptor (ESR1), and estradiol (E2) expressions were also assessed. Cell proliferation was evaluated using the CCK‐8 method. During T‐HESC decidualization, CYP27B1 expression significantly increased by Day 4, peaking on Day 8, whereas VDR expression increased progressively, and CYP24A1 levels remained stable. A high concentration of vitamin D significantly upregulated PRL and IGFBP1 transcription, increased CYP19 and VDR expression, elevated E2 and PRL secretion, and promoted ESC proliferation. VDR knockdown inhibited ESC decidualization, reducing PRL, IGFBP1, ESR1, and CYP19 expression, whereas VDR overexpression enhanced these markers. ChIP‐qPCR analysis demonstrated that VDR directly binds to the promoter regions of CYP19 and ESR1 in HESC. Vitamin D treatment significantly upregulated the expression of PRL, IGFBP1, CYP27B1, VDR, CYP19, and ESR1 in primary HESC on Day 8 of decidualization. These findings suggest that vitamin D promotes ESC decidualization in a dose‐ and time‐dependent manner via a VDR‐mediated mechanism, with estrogen signaling potentially playing a key role.

## 1. Introduction

Couples of childbearing age and pregnant women often experience vitamin D deficiency or insufficiency, a widespread public health concern [1–3]. Vitamin D is primarily synthesized in the skin upon sunlight exposure, with a smaller portion obtained from dietary sources. It functions as a steroid hormone with progesterone‐like activity [4, 5]. The predominant circulating form of vitamin D, 25‐hydroxyvitamin D (25(OH)D), is converted to its active form, 1,25‐dihydroxyvitamin D (1,25(OH)_2_D), by 1α‐hydroxylase (CYP27B1) in the kidneys and extrarenal tissues. It exerts its effects by binding to the vitamin D receptor (VDR) [6]. VDR, a member of the steroid receptor family, is widely expressed in various tissues of the female reproductive system, including the ovaries, granulosa cells, endometrium, and placenta [7].

In the field of reproductive health, vitamin D has gained significant research interest and potential for clinical application [8, 9]. Numerous studies have suggested that vitamin D may influence folliculogenesis, ovarian steroidogenesis, and endometrial receptivity, indicating its crucial role in regulating reproductive physiology [10, 11]. Our previous research supports the hypothesis that the vitamin D/VDR system plays an important role in the formation of endometrial receptivity [12]. Interestingly, we also found that the expression of CYP27B1 protein was significantly positively correlated with CYP19 during the implantation window of the endometrium. The CYP19 gene encodes aromatase, an enzyme of the cytochrome P450 family that catalyzes the final step in the conversion of androgens to estrogens [13].

Human endometrial decidualization is a key process regulating endometrial receptivity and is essential for the establishment of pregnancy. This process occurs during each menstrual cycle when endometrial stromal cells (ESCs) proliferate and differentiate into specialized decidual cells [14]. Decidualized ESC significantly enhances the secretion of factors such as prolactin (PRL) and insulin‐like growth factor–binding protein 1 (IGFBP1) [15]. This process is tightly regulated by estradiol (E2) and progesterone, which are derived from the ovary. E2 has been known to play a crucial role in progesterone receptor expression and promoting ESC differentiation [16, 17]. As demonstrated by Gibson et al., the decidualization of ESC significantly alters E2 metabolism within the tissue, with increased aromatase activity and expression, creating an E2‐dominated estrogen microenvironment that may enhance endometrial receptivity [18–20].

This study aims to investigate the role of the vitamin D/VDR system in promoting ESC decidualization in vitro and its underlying mechanisms. The research focus is on characterizing the expression of the vitamin D/VDR system, assessing its impact on decidualization markers, and elucidating its role in enhancing decidualization through CYP19‐mediated regulation of the estrogen microenvironment.

## 2. Materials and Methods

### 2.1. Materials

The reagents listed below were used in this investigation: β‐E2 was purchased from Sigma‐Aldrich, whereas calcitriol and medroxyprogesterone acetate were obtained from APExBIO. Carbon‐adsorbed fetal bovine serum (FBS) was provided by Biological Industries. Sodium bicarbonate, sodium pyruvate solution, ITS liquid media supplement, and N6,2′‐O‐dibutyryladenosine 3′,5′‐cyclic monophosphate sodium salt were all purchased from Sigma‐Aldrich. The DMEM/F‐12 medium with L‐glutamine, sodium pyruvate, and 15 mm HEPES was provided by Meilunbio. Puromycin was obtained from Solarbio, and penicillin–streptomycin solution was purchased from Gibco.

### 2.2. T‐Human ESC (HESC) Culture Conditions

The T‐HESCs were acquired from ATCC (CRL‐4003), and the cells were thawed according to the instructions provided by the manufacturer. The DMEM/F‐12 medium used for culturing T‐HESCs was supplemented with 10% charcoal‐stripped FBS, 50 U/mL penicillin, and 50 μg/mL streptomycin. To induce in vitro differentiation, cells were cultured in MEM/F‐12 medium containing 0.5 mm db‐cAMP, 1 μm medroxyprogesterone acetate, and 10 nm β‐E2, with an additional 2% charcoal‐stripped FBS. The cells were cultured in 25‐cm^2^ flasks at 37°C in a humidified atmosphere consisting of 95% air and 5% CO_2_ for 8 days. Subculturing was performed when the cells reached approximately 90% confluency, with the medium and differentiation cocktail refreshed every 48 h.

### 2.3. Primary Cultures of HESC

Endometrial tissue was obtained from infertile women undergoing hysteroscopy. All patients had regular menstrual cycles and were ≤ 40 years old. Tissue was collected during the proliferative phase, placed in HBSS with 1% penicillin–streptomycin, and transported to the culture lab for digestion. Pathological examination was performed to exclude abnormalities. The tissue was digested with 2 mg/mL collagenase in DMEM/F‐12 medium at 37°C for 1 h. After centrifugation and washing, cells were filtered through a 40‐μm cell strainer to isolate stromal cells and subsequently cultured for 6 h to allow adhesion. Nonadherent cells were removed, and the adherent fraction was maintained for further proliferation. To induce in vitro differentiation, cells were cultured in MEM/F‐12 with 0.5 mm db‐cAMP, 1 μm medroxyprogesterone acetate, and 2% charcoal‐stripped FBS. Cells were incubated in 25‐cm^2^ flasks at 37°C, 95% air/5% CO_2_ for 8 days, with medium replaced every 24 h. In the treatment group, 10^−7^ M 1,25(OH)_2_D was added; in the control group, the same volume of ethanol as solvent for 1,25(OH)_2_D was added. The concentration was based on previous studies [21].

### 2.4. Silence or Overexpression of VDR in T‐HESCs

To regulate VDR expression in T‐HESCs, VDR cDNA plasmids and siRNA targeting VDR were utilized. Transfections were performed using Lipofectamine 2000 (Invitrogen, USA) following the manufacturer’s protocol for 48 h. The siRNA sequences were as follows: sense, 5′‐GAC​UCU​CGC​UCU​UCU​ACU​UTT‐3′; antisense, 5′‐AAG​UAG​AAG​AGC​GAG​AGU​CTT‐3′. Transfection efficiency was confirmed via quantitative qPCR and Western blot, using GAPDH as an internal control. After transfection, cells were exposed to 100 ng/mL 1,25(OH)_2_D in DMEM/F‐12 medium supplemented with 2% charcoal‐stripped FBS for 8 days to induce differentiation, as described in Section 2.2.

### 2.5. Immunofluorescence

T‐HESCs were seeded into 24‐well plates containing coverslips at a density of 5 × 10^4^ cells/well. The cells were then treated with differentiation and proliferation media, respectively. Standard immunofluorescence staining was performed on the coverslips. 0.1 μmol/L Acti‐Stain 488 Phalloidin (Cytoskeleton, USA) was used to stain F‐actin, and nuclei were counterstained with DAPI (0.2 μg/mL) for 10 min. Fluorescence images were captured to visualize the cytoskeleton and nuclei. Imaging and processing were conducted using consistent microscopy settings to ensure data reliability and comparability. The proportion of double‐nucleated cells was visually estimated from DAPI‐stained images using ImageJ software. For primary HESC identification, cells were seeded onto coverslips in 24‐well plates at a density of 5 × 10^4^ cells/well. After 24 h of culture, the cells were fixed with 4% paraformaldehyde and subjected to immunofluorescence staining using antivimentin (1:100, Proteintech, 10366‐1‐AP) and anticytokeratin 8 (1:100, Proteintech, 10384‐1‐AP) as primary antibodies. Goat antirabbit IgG secondary antibodies (Abcam, ab150077; Proteintech, SA00013‐4; 1:400) were used. DAPI (0.2 μg/mL) was applied for nuclear counterstaining.

### 2.6. CCK‐8 Assay

Cell proliferation was assessed using the CCK‐8 assay according to the manufacturer’s guidelines. After trypsinization and cell counting, cells were seeded into a 96‐well plate at a density of 3000 cells per 100 μL. Each well received 10 μL of CCK‐8 solution and was incubated for 2 h. Absorbance at 450 nm was measured using a microplate reader. Proliferation rates were recorded every 24 h. Five replicate wells were prepared for each time point, and the experiment was repeated three times to ensure reliability.

### 2.7. RNA Extraction, Reverse Transcription, and Real‐Time qPCR

Total RNA was extracted using the TRIzol total RNA method. The PrimeScript RT Reagent Kit (Takara Bio) was used to perform reverse transcription with 2 μg of RNA. qPCR was conducted on an AB7800 detection system. cDNA detection was performed according to the guidelines provided by the SYBR Premix Ex Taq Kit (Takara). Under the conditions of 95°C predenaturation for 30 s, followed by 40 cycles of 95°C for 5 s and 60°C for 31 s, each sample reaction was conducted in duplicate. The expression levels of the target genes were normalized using GAPDH as a reference. The primers used for real‐time PCR were as follows: PRL, 5′‐CTT​CAT​TCC​AGA​AGT​ACC​CT‐3′ (forward) and 5′‐TCT​TTC​CCA​GAT​ATT​GGC​TT‐3′ (reverse); IGFBP1, 5′‐GAG​ATA​ACT​GAG​GAG​GAG‐3′ (forward) and 5′‐CCA​AAG​GAT​GGA​ATG​ATC‐3′ (reverse); CYP27B1, 5′‐AGA​GTT​GCT​ATT​GGC​GGG​AG‐3′ (forward) and 5′‐GGA​GTG​CTG​TCT​GGA​CTT​CG‐3′ (reverse); CYP24A1, 5′‐TGG​GTT​CCT​TTG​AGT​CGG​TG‐3′ (forward) and 5′‐TCC​ACG​GTT​TGA​TCT​CCA​GC‐3′ (reverse); VDR, 5′‐TGG​AGA​CTT​TGA​CCG​GAA​CG‐3′ (forward) and 5′‐GAA​GCC​TTT​GCA​GCC​TTC​AC‐3′ (reverse); CYP19, 5′‐GAC​GTC​GCG​ACT​CTA​AAT​TG‐3′ (forward) and 5′‐ACC​CGG​TTG​TAG​TAG​TTG​CAG‐3′ (reverse); estrogen receptor (ESR1), 5′‐GGG​AAG​TAT​GGC​TAT​GGA​ATC​TG‐3′ (forward) and 5′‐TGG​CTG​GAC​ACA​TAT​AGT​CGT​T‐3′ (reverse); and GAPDH (internal control), 5′‐AAG​GTC​ATC​CCT​GAG​CTG​AAC‐3′ (forward) and 5′‐ACG​CCT​GCT​TCA​CCA​CCT​TCT‐3′ (reverse). The 2−ΔCT method was used to calculate relative mRNA levels.

### 2.8. Protein Extraction and Western Blotting

Using a Total Protein Extraction Kit by Beyotime, total protein was extracted, and the concentration was assessed with Solarbio’s BCA protein concentration assay kit as per the guidelines. The separation of 40 μg of protein was achieved through 10% SDS‐PAGE, followed by transfer to a PVDF membrane. The membrane was blocked at room temperature with 5% BSA in PBS for 2 h, followed by an overnight incubation with primary antibodies at 4°C. The antibodies used in this study included CYP27B1 (ABclonal), CYP24A1 (ABclonal), VDR (Abcam), CYP19 (Abcam), ESR1 (Abcam), and GAPDH (Abcam). After washing with PBS, membranes were treated with a secondary antibody conjugated to HRP and then washed again using PBS. A calibrated Bio‐Rad GS 800 densitometer was used to scan the films, and ImageJ software was employed for signal analysis.

### 2.9. ELISA

Hormone concentrations were measured using ELISA. Cell supernatants from five groups were collected at different time points. The levels of E2 and PRL in the supernatants were determined using human PRL and E2 ELISA kits (Cloud‐Clone) following the manufacturer’s guidelines. The details of the ELISA kits are provided in the Supporting Information (Table S1).

### 2.10. Chromatin Immunoprecipitation (ChIP)

ChIP was performed to investigate the binding of VDR to the promoters of CYP19 and ESR1. Cells were crosslinked with 1% formaldehyde (Sigma, Cat. No. f8775), and the reaction was quenched with 1 × glycine (Sigma, Cat. No. 5652). After cell lysis and nuclease digestion, chromatin was immunoprecipitated using VDR‐specific antibody (Proteintech, Cat. No. 67192‐1), positive control H3K27ac antibody (Abcam, Cat. No. ab4729), and negative control IgG antibody (CST, Cat. No. 2729). The DNA–protein complexes were captured with Protein A/G beads (Pierce, Cat. No. 26156), washed, and eluted. The purified DNA was analyzed by qPCR to quantify the binding of VDR to the target gene promoters.

### 2.11. Statistical Analysis

The statistical analyses were carried out using GraphPad Prism 9.0 software (GraphPad Software Inc., La Jolla, CA, USA). ANOVA and Student’s *t*‐test were applied to compare continuous variables, with a *p*‐value less than 0.05 regarded as significant. Intergroup differences were assessed by ANOVA followed by Tukey’s multiple comparisons test. A two‐tailed adjusted *p*‐value of less than 0.05 was considered statistically significant. Data were shown as mean ± SD from triplicate technical experiments.

## 3. Results

### 3.1. Establishment of an In Vitro Decidualization Model

An in vitro decidualization model was successfully established using T‐HESC cell lines. Cells were cultured in differentiation medium for 8 days, and marker expression was analyzed at multiple time points. PRL mRNA levels significantly increased by Day 2, peaking at Day 8 (Figure 1(a)). IGFBP‐1 mRNA expression rose significantly from Day 4, reaching its maximum on Day 8 (Figure 1(b)). Immunofluorescence staining of F‐actin revealed typical decidual stromal cell morphology, confirming successful differentiation (Figure 1(c)).

FIGURE 1Establishment of the in vitro decidualization model. Relative mRNA levels of (a) PRL and (b) IGFBP‐1 measured at Days 0, 2, 4, and 8 during decidualization using RT‐qPCR. (c) Immunofluorescence analysis of F‐actin (green) and DAPI‐stained nuclei (blue), showing the typical morphology of decidual stromal cells at Day 8. The images captured at a magnification of 200x serve as representative visual representations of three distinct and separate samples. Scale bar = 200 μm. Data were presented as mean ± SD. ^∗^ *p* < 0.05.

**Figure:**
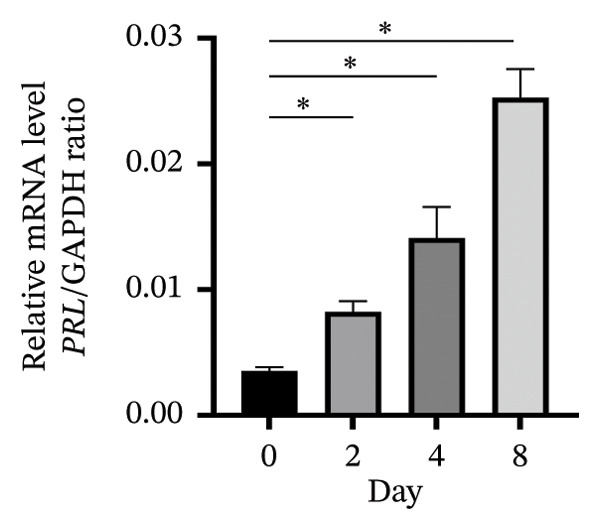
(a)

**Figure:**
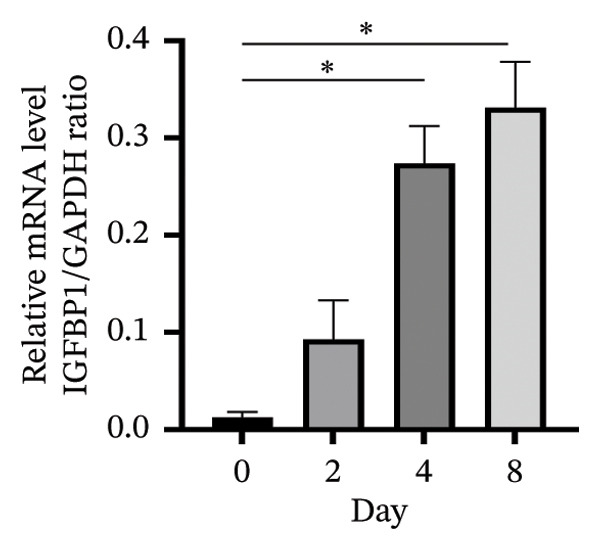
(b)

**Figure:**
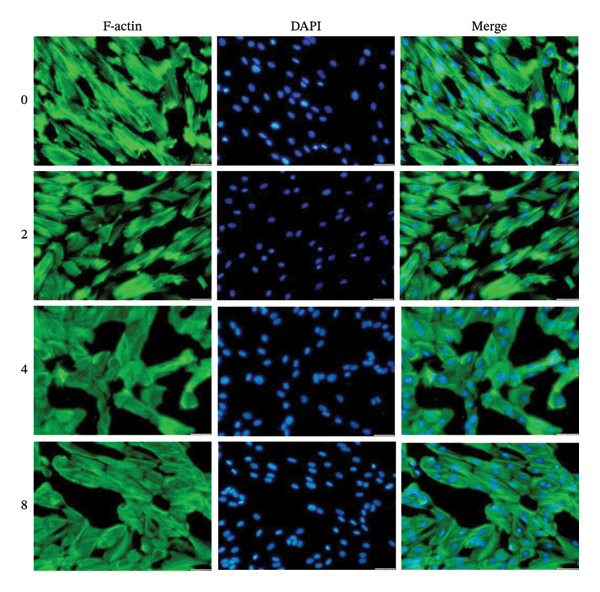
(c)

### 3.2. Identification and Decidualization Induction of Primary HESC

To identify primary HESC, immunofluorescence staining for cytokeratin and vimentin was performed 1 day after seeding. Cytokeratin was absent, whereas vimentin showed strong positive expression, confirming the cells as ESCs with > 95% purity (Figure 2(a)). A successful in vitro decidualization model was established using primary HESC. The cells were cultured in differentiation medium for 8 days, with PRL levels analyzed at multiple time points. PRL concentration significantly increased by Day 4 and peaked on Day 8 (Figure 2(c)). Optical microscopy confirmed decidualization. Undifferentiated cells showed a spindle‐shaped, fibroblast‐like morphology, whereas decidualized cells displayed an enlarged, pebble‐like, sawtooth‐shaped morphology (Figure 2(b)).

FIGURE 2Characterization and decidualization of primary HESC. (a) Immunofluorescence staining of vimentin (red) in primary HESC, confirming the stromal phenotype with > 95% purity. Cytokeratin staining was absent, indicating minimal epithelial contamination (40x, scale bar = 100 μm). (b) Morphological changes in primary HESC after 8 days of differentiation. Undifferentiated cells displayed a spindle‐shaped, fibroblast‐like morphology, whereas decidualized cells exhibited enlarged, pebble‐like, sawtooth‐shaped morphology (100x, scale bar = 300 μm). (c) PRL expression during in vitro decidualization. PRL concentration significantly increased on Day 4 and peaked on Day 8.

**Figure:**
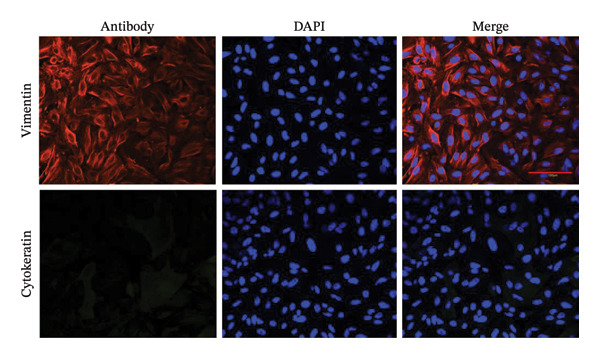
(a)

**Figure:**
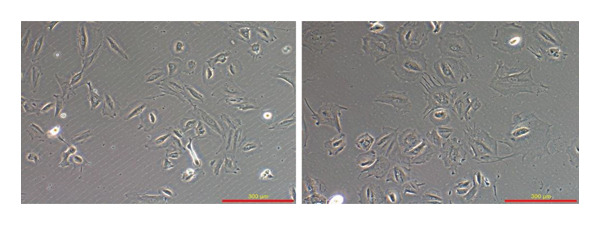
(b)

**Figure:**
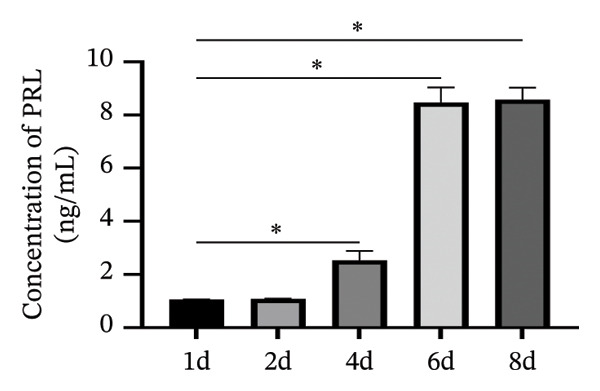
(c)

### 3.3. Expression of the Vitamin D/VDR System in Human Decidualized ESC

The role of the vitamin D/VDR system in decidualization was investigated using an in vitro model. CYP27B1, responsible for the synthesis of active vitamin D, gradually increased during decidualization, with significant mRNA and protein elevation by Day 4, peaking on Day 8 (Figures 3(a), 3(d)). In contrast, CYP24A1, involved in the inactivation of vitamin D, showed no significant change throughout the process (Figures 3(b), 3(d)). VDR expression at both mRNA and protein levels increased significantly, reaching its maximum on Day 8 (Figures 3(c), 3(d)).

FIGURE 3Expression of the vitamin D/VDR system in human decidualized ESC. Relative mRNA levels of (a) CYP27B1, (b) CYP24A1, and (c) VDR measured at Days 0, 2, 4, and 8 during decidualization using RT‐qPCR. (d) Western blot analysis of CYP27B1, CYP24A1, and VDR protein levels. Data were presented as mean ± SD. ^∗^ *p* < 0.05.

**Figure:**
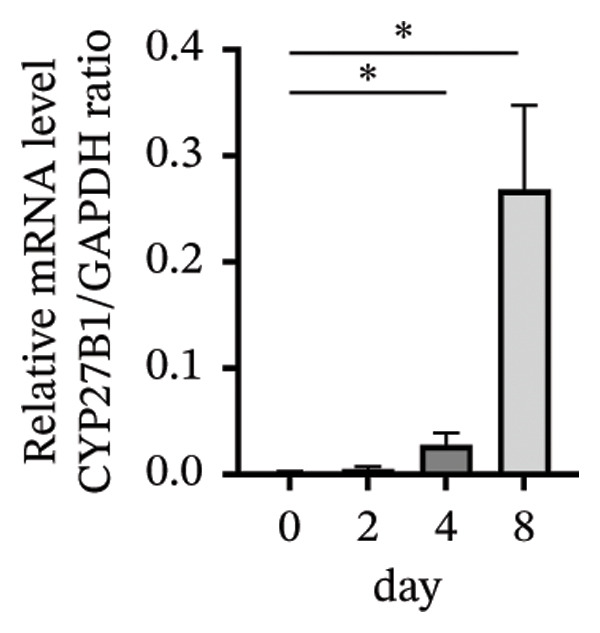
(a)

**Figure:**
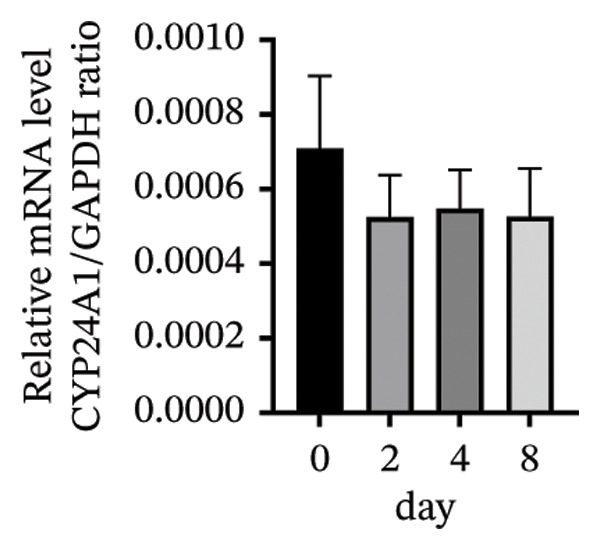
(b)

**Figure:**
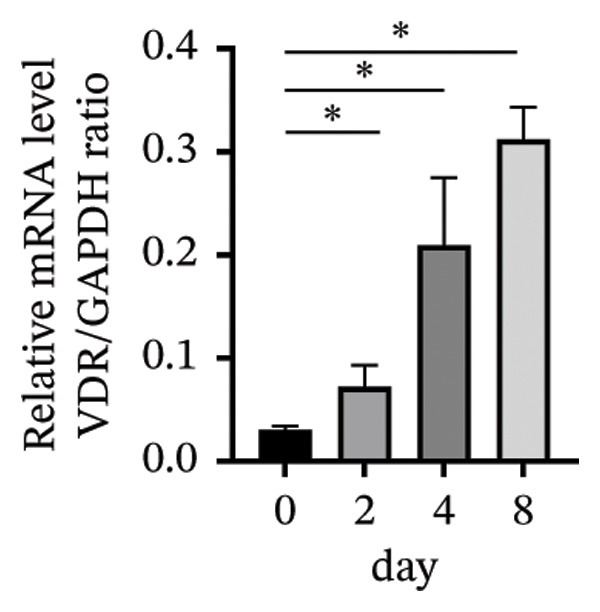
(c)

**Figure:**
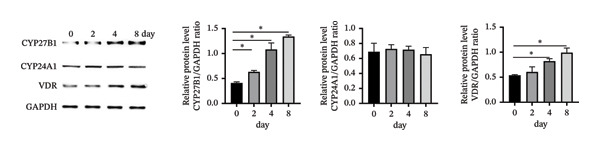
(d)

### 3.4. Effect of Vitamin D/VDR on Human ESC Decidualization

#### 3.4.1. Effects of Different Vitamin D Levels on T‐HESC Decidualization

To investigate the role of vitamin D in decidualization, T‐HESCs were treated with 0, 10, 50, and 100 ng/mL of 1,25(OH)_2_D and cultured in differentiation medium for 4 and 8 days. As vitamin D concentrations increased, PRL mRNA levels significantly increased, whereas IGFBP1 levels were markedly elevated at concentrations ≥ 50 ng/mL (Figures 4(a), 4(c)). ELISA results showed a positive relationship between vitamin D levels and the secretion of E2 and PRL (Figures 5(a), 5(b)). CCK‐8 assays showed that higher vitamin D levels significantly enhanced cell proliferation (Figure 5(c)). Similarly, CYP19 and VDR expression increased significantly at ≥ 50 ng/mL on both Day 4 and Day 8, following a consistent trend (Figures 4(a), 4(b), 4(c), 4(d)).

FIGURE 4Effects of vitamin D concentrations on decidualization markers, CYP19, and VDR in T‐HESC. Relative mRNA levels of PRL, IGFBP1, CYP19, and VDR at (a) Day 4 and (c) Day 8 following treatment with 0, 10, 50, and 100 ng/mL of 1,25(OH)_2_D (vitamin D), measured using RT‐qPCR. Western blot analysis of CYP19 and VDR protein expression at (b) Day 4 and (d) Day 8. Data were presented as mean ± SD. ^∗^ *p* < 0.05.

**Figure:**
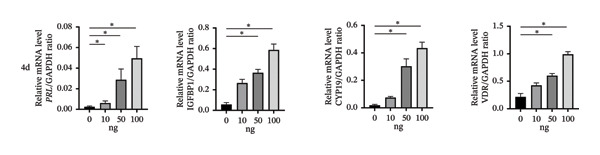
(a)

**Figure:**
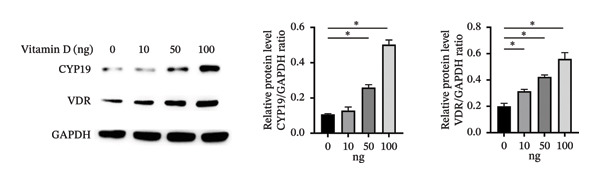
(b)

**Figure:**
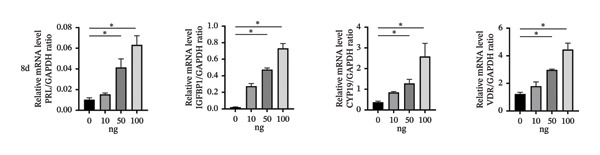
(c)

**Figure:**
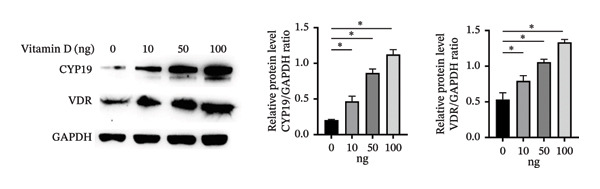
(d)

FIGURE 5Effects of vitamin D concentrations on E2 and PRL secretion and cell proliferation during endometrial decidualization. ELISA measurement of E2 and PRL secretion on (a) Day 4 and (b) Day 8 following treatment with 1,25(OH)_2_D at 0, 10, 50, and 100 ng/mL. (c) CCK‐8 assay assessing cell proliferation. Data were presented as mean ± SD. ^∗^ *p* < 0.05.

**Figure:**
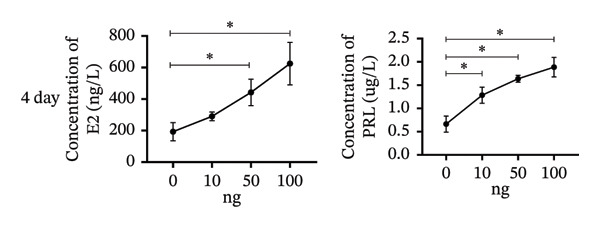
(a)

**Figure:**
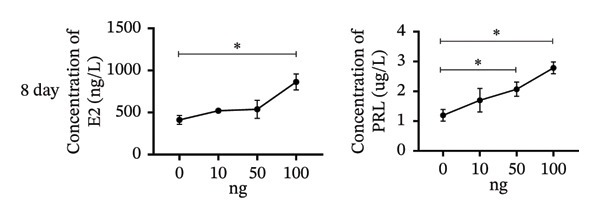
(b)

**Figure:**
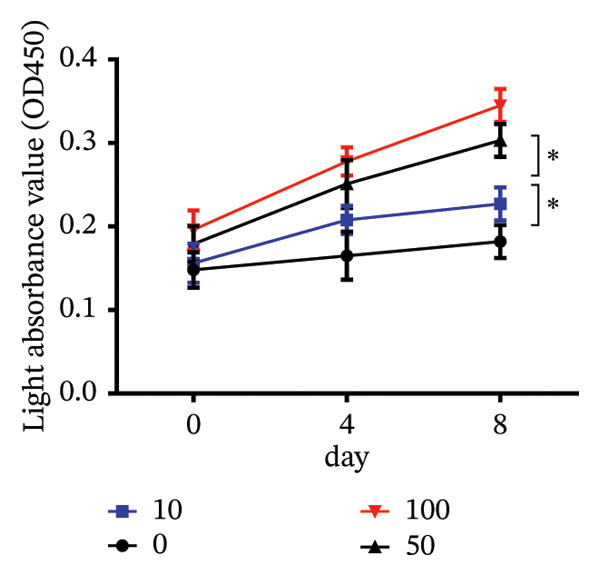
(c)

#### 3.4.2. Effects of Vitamin D on Primary HESC Decidualization

To investigate vitamin D’s impact on primary HESC decidualization, cells were treated with 10^−7^ M 1,25(OH)_2_D or a control and cultured for 4 and 8 days. RT‐qPCR analyzed mRNA levels of PRL, IGFBP1, CYP27B1, CYP24A1, VDR, CYP19, and ESR1. On Day 4, CYP19 and ESR1 levels were significantly higher with vitamin D treatment (Figures 6(f), 6(g)). By Day 8, PRL, IGFBP1, CYP27B1, VDR, CYP19, and ESR1 levels were notably increased (Figures 6(h), 6(i), 6(j), 6(l), 6(m), 6(n)), whereas CYP24A1 showed no significant change.

FIGURE 6Effects of vitamin D on the expression of decidualization markers and estrogen‐related genes in primary HESC. Relative mRNA levels of PRL, IGFBP1, CYP27B1, CYP24A1, VDR, CYP19, and ESR1 were determined by RT‐qPCR following treatment with 10^−7^ M 1,25(OH)_2_D or vehicle control for (a–g) 4 days and (h–n) 8 days. Data were presented as mean ± SD. ^∗^ *p* < 0.05.

**Figure:**
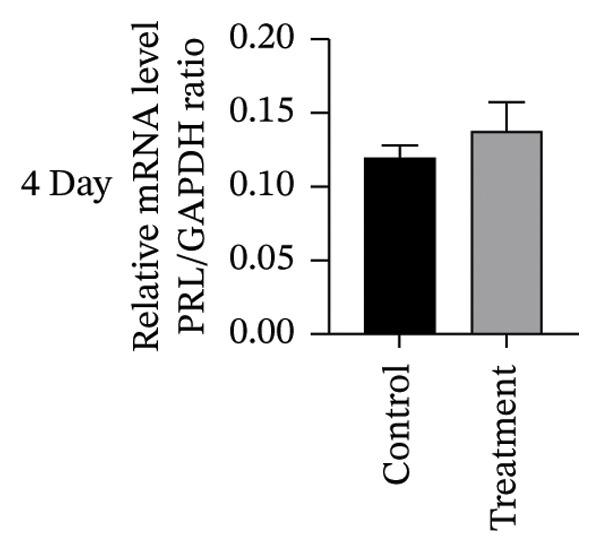
(a)

**Figure:**
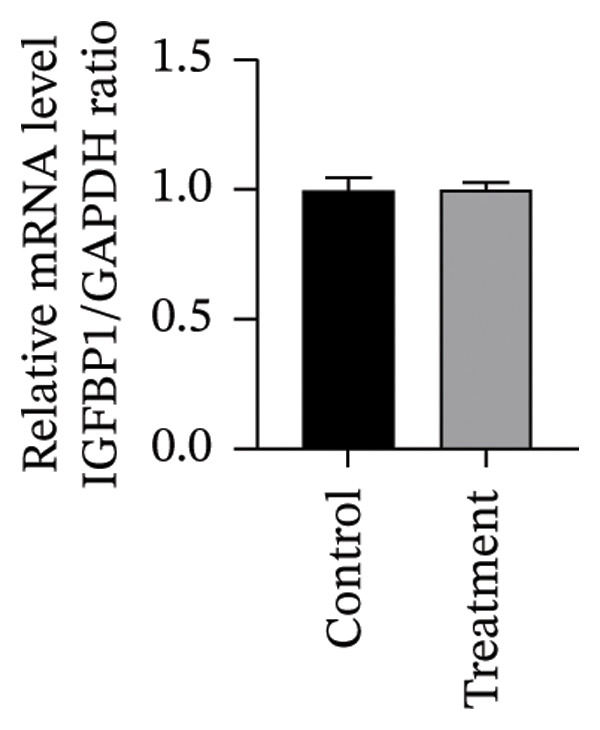
(b)

**Figure:**
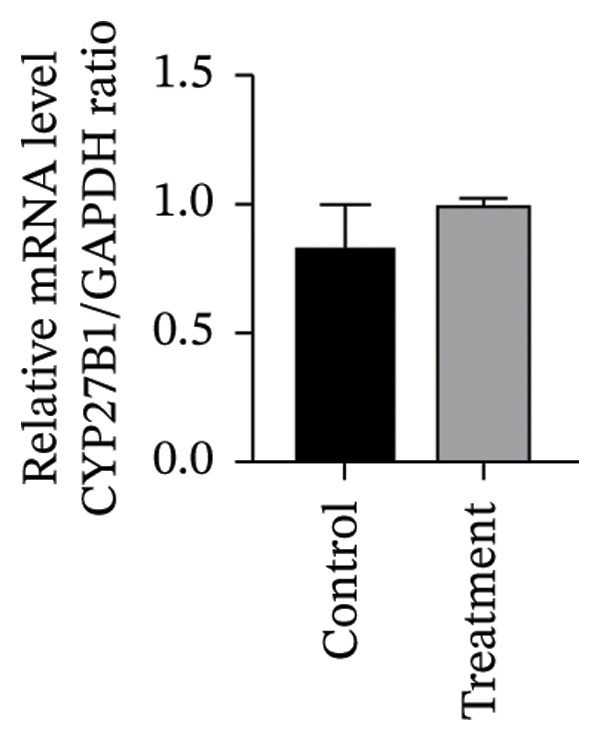
(c)

**Figure:**
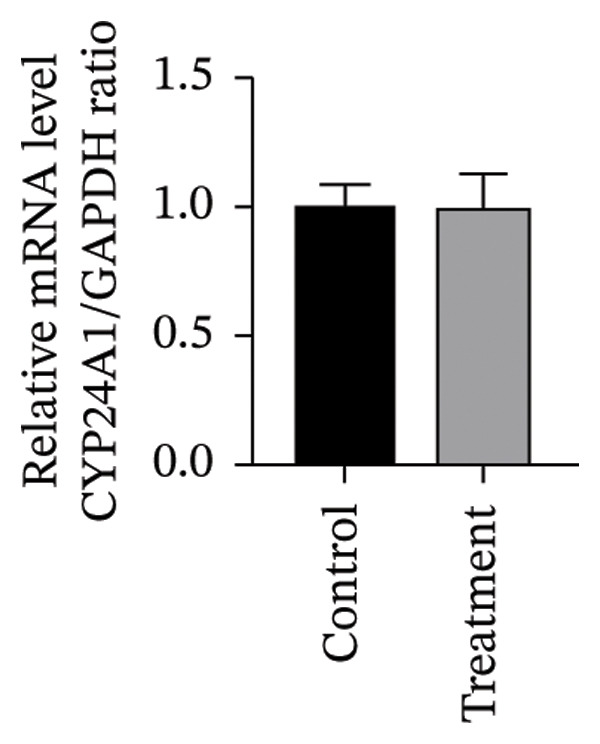
(d)

**Figure:**
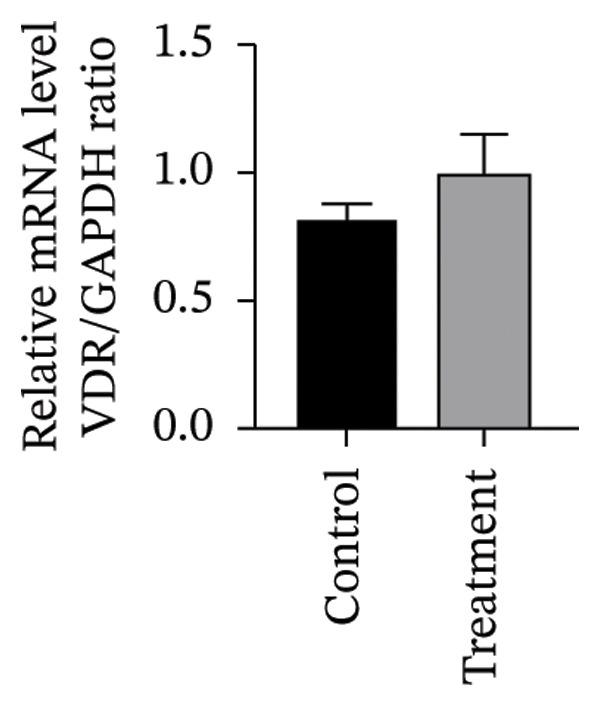
(e)

**Figure:**
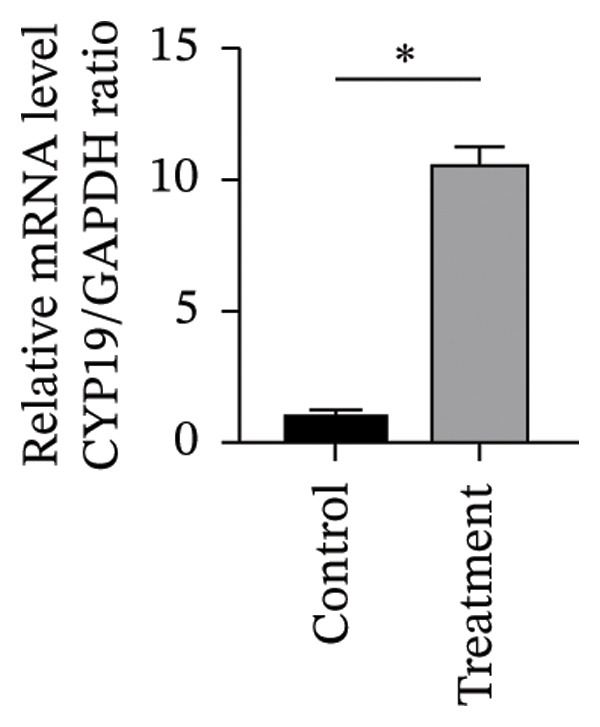
(f)

**Figure:**
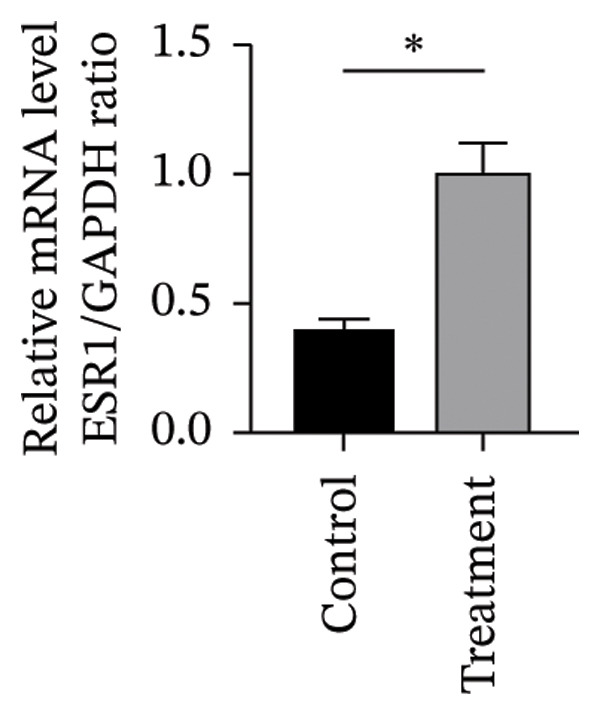
(g)

**Figure:**
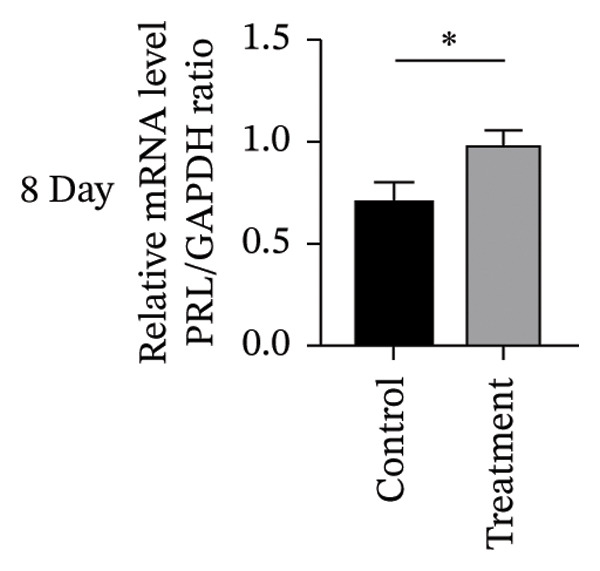
(h)

**Figure:**
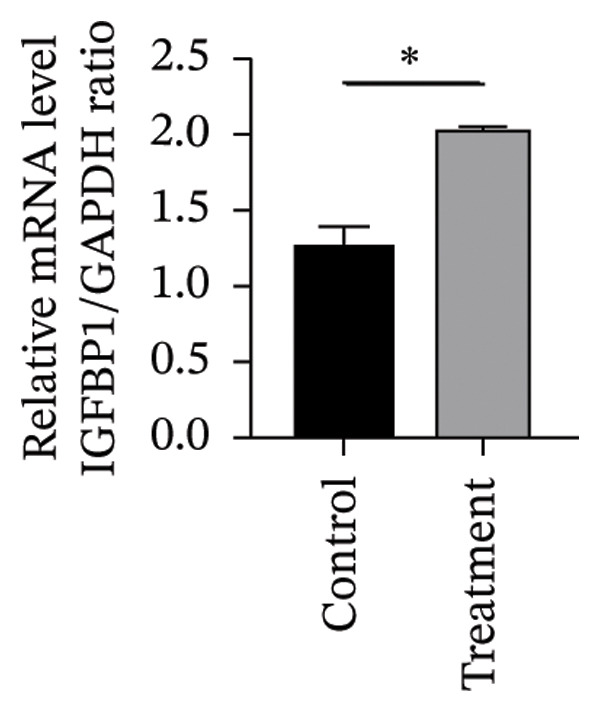
(i)

**Figure:**
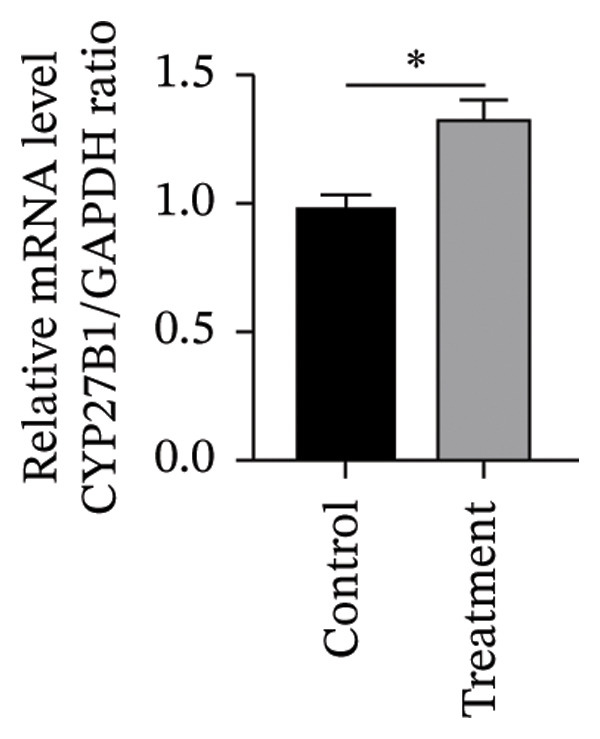
(j)

**Figure:**
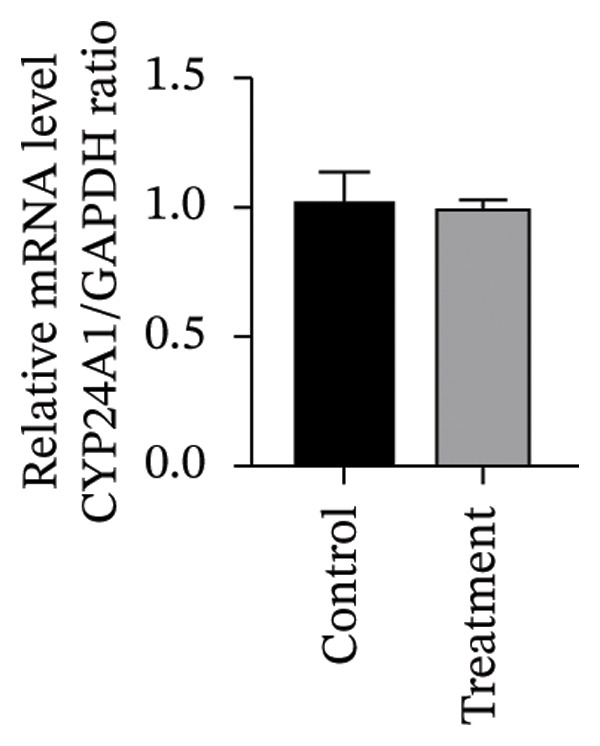
(k)

**Figure:**
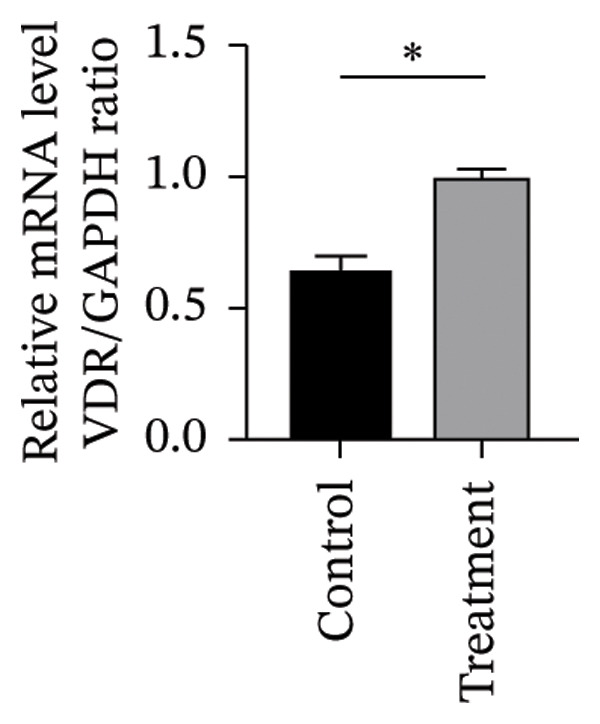
(l)

**Figure:**
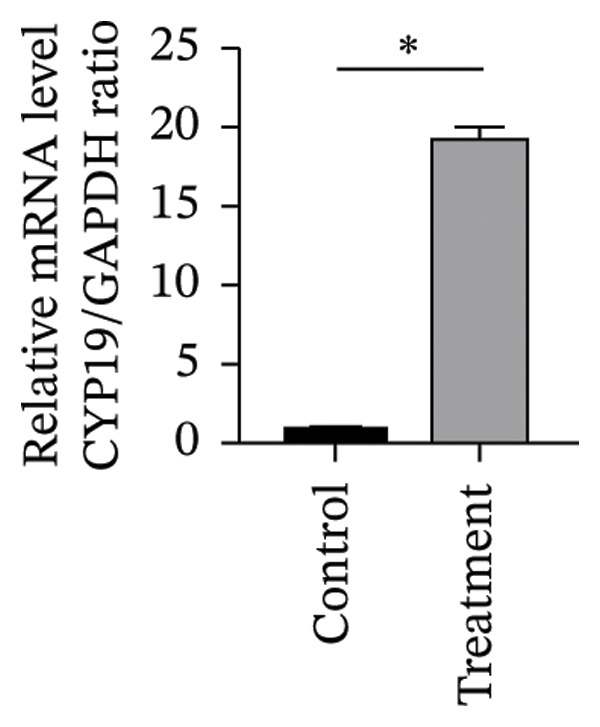
(m)

**Figure:**
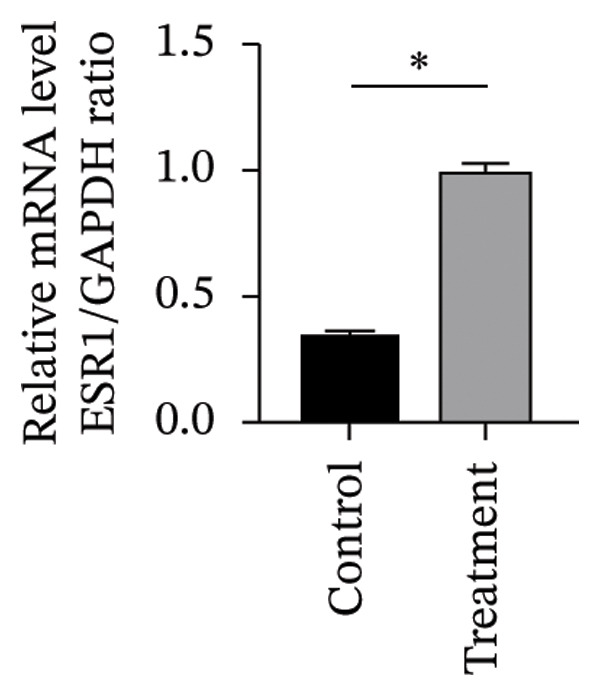
(n)

#### 3.4.3. The Effects of VDR Expression on the Decidualization of HESC

To investigate the molecular mechanisms through which vitamin D regulates decidualization, transiently transfected T‐HESC were generated with VDR knockdown, overexpression, and respective controls. qPCR and Western blot confirmed that VDR knockdown significantly reduced VDR mRNA and protein levels, whereas overexpression markedly increased them (Figure 7). Cells were subsequently cultured in differentiation medium containing 100 ng/mL vitamin D for 8 days. VDR knockdown significantly reduced PRL and IGFBP1 mRNA levels, whereas overexpression significantly increased them (Figure 8(a)). Additionally, qPCR and Western blot analyses showed that VDR knockdown significantly downregulated ESR1 and CYP19 mRNA and protein levels, whereas overexpression significantly upregulated them (Figures 8(b), 8(c)). To clarify how VDR regulates CYP19 and ESR1 expression, a ChIP assay was conducted in T‐HESCs. The results showed that VDR directly binds to promoters, activating CYP19 and ESR1 transcription (Figure 9).

FIGURE 7Generation and validation of transiently transfected T‐HESC cell lines with modified VDR expression levels. (a) RT‐qPCR measured VDR mRNA levels in cells with VDR knockdown (si‐VDR) and overexpression (OV‐VDR) versus controls. (b) VDR protein levels evaluated by Western blot. Data were presented as mean ± SD. ^∗^ *p* < 0.05.

**Figure:**
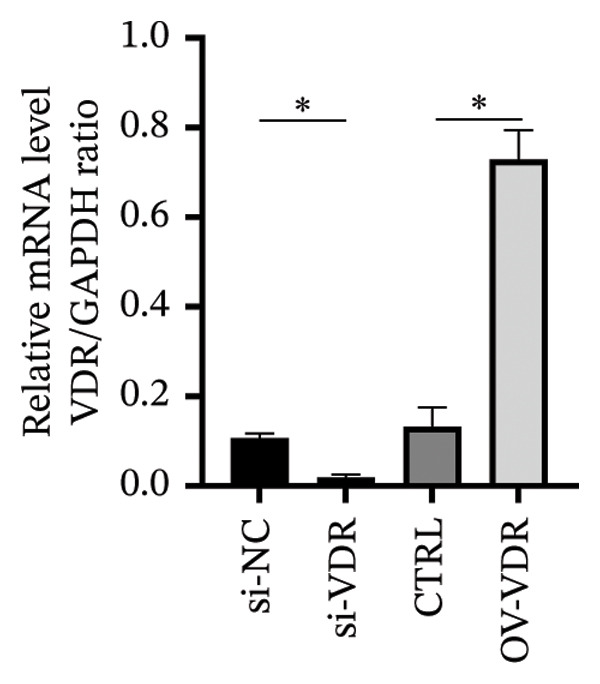
(a)

**Figure:**
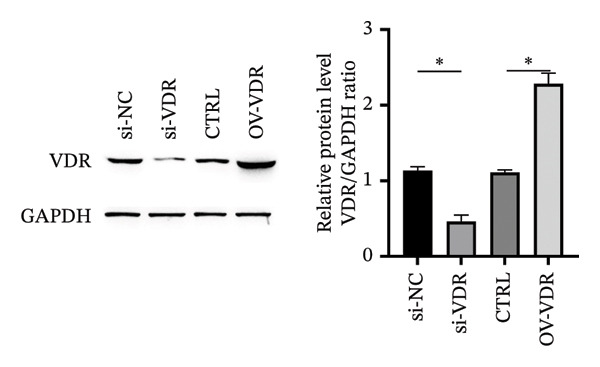
(b)

FIGURE 8Impact of VDR knockdown or overexpression on decidualization markers and ESR1/CYP19 expression in T‐HESC. (a) PRL and IGFBP1 mRNA levels after VDR knockdown (si‐VDR) or overexpression (OV‐VDR) via RT‐qPCR. (b) VDR, ESR1, and CYP19 mRNA levels via RT‐qPCR. (c) VDR, ESR1, and CYP19 protein levels via Western blot. Data were presented as mean ± SD. ^∗^ *p* < 0.05.

**Figure:**
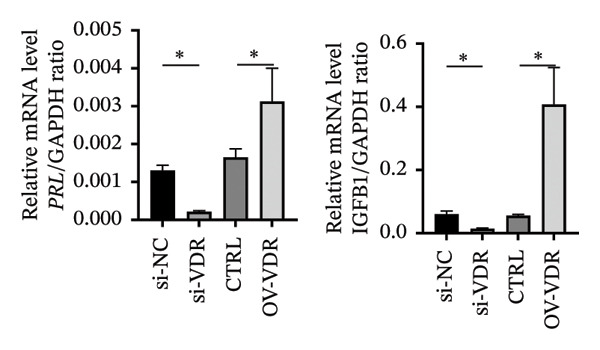
(a)

**Figure:**
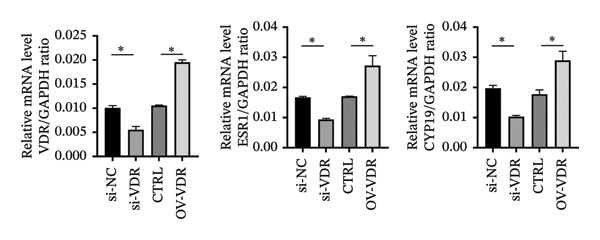
(b)

**Figure:**
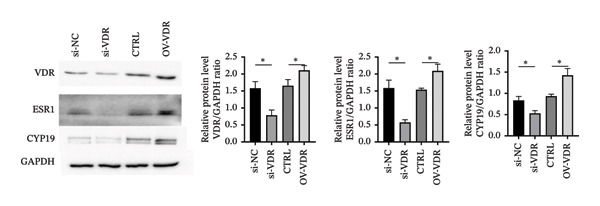
(c)

FIGURE 9ChIP assay demonstrates VDR’s direct binding to CYP19 and ESR1 promoters in HESC. (a) VDR antibody specificity is confirmed by a single band at 48–55 kDa, with GAPDH (36 kDa) as a control. (b) Chromatin fragmentation shows effective shearing with DNA fragments mostly between 100 and 200 bp. (c) qPCR indicates significant enrichment of CYP19 and ESR1 promoters in VDR‐immunoprecipitated chromatin, confirming direct VDR interaction. (d) Assay specificity is validated with a positive control (H3K27ac at GAPDH promoter) and a negative control (rabbit IgG at the same region).

**Figure:**
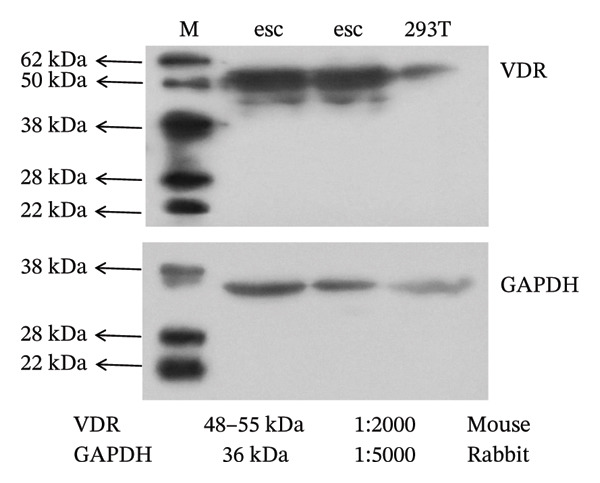
(a)

**Figure:**
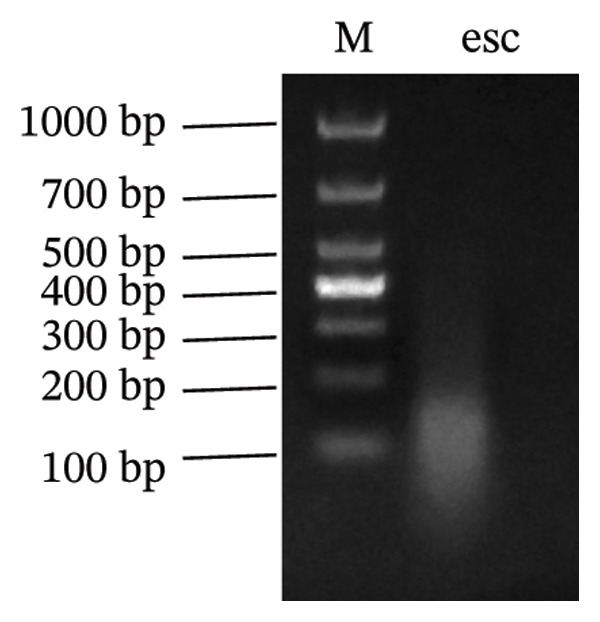
(b)

**Figure:**
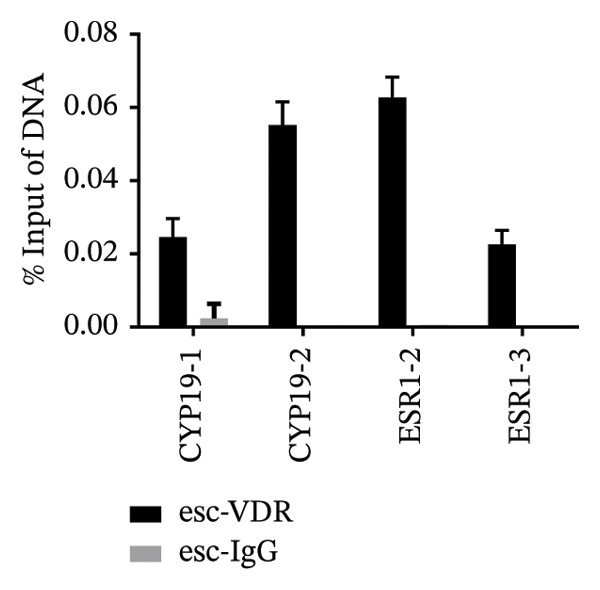
(c)

**Figure:**
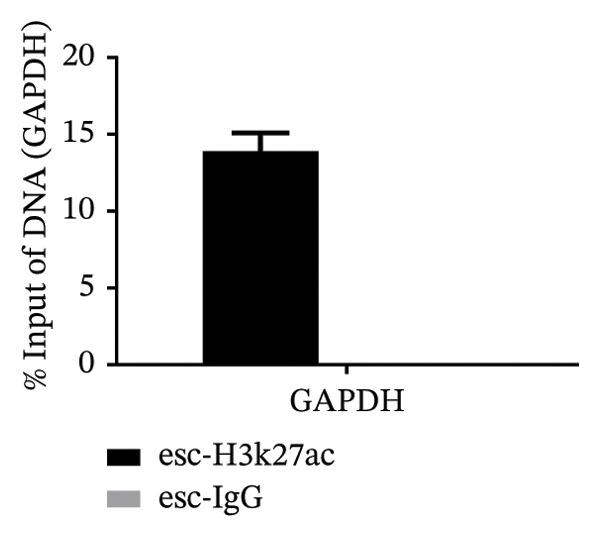
(d)

## 4. Discussion

Vitamin D deficiency is a global health concern, particularly in Asia, where it affects a large portion of the population [22]. In our previous study, we found that 28.3% of infertile women in China had vitamin D deficiency, which was associated with significantly lower clinical pregnancy and live birth rates among IVF patients, even after controlling for factors such as embryo quality [23]. These findings suggest a potential link between serum vitamin D levels and endometrial receptivity. We further discovered that human endometrium expresses VDR throughout the menstrual cycle and has the capacity to metabolize vitamin D, indicating that the endometrium is a target tissue for vitamin D [12]. It is well‐established that decidualization of ESC is a crucial aspect of endometrial receptivity [24]. In this study, we confirmed that the vitamin D/VDR system promotes the proliferation and decidualization of human ESC within an estrogen‐dominated microenvironment, as observed in our in vitro decidualization model.

Vitamin D metabolic enzymes are expressed in various female reproductive organs, indicating that female reproductive tissues play a significant role in vitamin D metabolism beyond the kidneys [25]. In the current study, we confirmed a novel finding that CYP27B1 is significantly upregulated during the decidualization of ESC, and VDR expression follows a similar trend. These results align with previous studies on human ESC and decidual stromal cells [26, 27], as well as studies on the expression of 1α‐hydroxylase in the pregnant porcine endometrium [28]. Given the role of CYP24A1 in vitamin D metabolism, we also investigated its expression during the decidualization process. However, no significant changes in CYP24A1 expression were observed in our study. Some researchers have found elevated CYP24A1 expression in uterine leiomyomas compared to adjacent normal myometrial tissues [29], whereas no difference was observed in the endometrium across menstrual cycle stages [30]. These findings suggest that local vitamin D metabolism in the uterus varies under different physiological and pathophysiological conditions and between tissues. Overall, vitamin D activation appears to be enhanced during ESC decidualization.

Normal endometrial decidualization is vital for successful embryo implantation and pregnancy establishment. To better accommodate embryo implantation, ESCs transform into large, round, multinucleated cells with abundant cytoplasm, called decidual stromal cells. This process, narrowly termed as “decidualization,” is necessary for the formation of a receptive endometrium [31]. Decidual stromal cells exhibit significant expression of secreted proteins, including PRL and IGFBP‐1, which are well‐established markers of decidualization. In this study, we found that vitamin D effectively increased the concentrations of PRL and IGFBP‐1 in decidualized ESC in a time‐ and dose‐dependent manner. Results from VDR interference by siRNA and VDR overexpression experiments indicated that vitamin D–regulated ESC decidualization was dependent on VDR. To our knowledge, this is the first study to report this finding, consistent with Hosseinirad’s recent study, which showed that 1,25(OH)_2_D_3_ significantly induced PRL concentration and enhanced ultrastructural changes in decidualized ESC [32]. Additionally, we observed that higher concentrations of vitamin D significantly promoted the proliferation of decidualized ESC, a critical step in the decidualization process. This finding is consistent with evidence that ESC proliferation is essential for transforming the endometrium into a receptive state for embryo implantation [33, 34]. This molecular mechanism could explain clinical observations that vitamin D supplementation increases endometrial thickness in infertile women [35], further supporting its potential therapeutic value in improving endometrial receptivity.

Studies have shown that, during decidualization, increased aromatase expression and activity (encoded by CYP19) create an estrogen‐dominated microenvironment in the endometrium [18]. E2 plays a pivotal role in the initial stages of decidualization [16]. The implantation window is characterized by peak E2 levels [20], which stimulate the progesterone receptor transcription. Furthermore, E2 enhances the endometrium’s response to progesterone, ultimately inducing ESC decidualization through the coordinated actions of progesterone and cAMP [17]. In our in vitro model, we demonstrated that high concentrations of vitamin D significantly upregulated the expression levels of CYP19. Additionally, the transcription levels of PRL and IGFBP1, markers of decidualization, were also significantly increased. Further investigation revealed that silencing VDR notably decreased the expression levels of CYP19 and ESR1, thereby impairing ESC decidualization. Conversely, VDR overexpression significantly reversed the inhibitory effects on CYP19 and ESR1 expression levels and ESC decidualization. ChIP‐qPCR analysis showed that VDR directly binds to the promoter regions of CYP19 and ESR1 in HESC, thereby showing that vitamin D/VDR promotes decidualization by regulating estrogen‐related genes.

Our findings strongly suggest that vitamin D enhances HESC decidualization in vitro through VDR‐dependent mechanisms. In particular, we found that vitamin D upregulated CYP19 expression by interacting with VDR, potentially modulating local E2 levels. Moreover, vitamin D enhanced the expression of ESR1 and increased the sensitivity of ESC to E2, ultimately facilitating the decidualization process through the ESR1 signaling pathway. Interestingly, our findings indicate that sufficient vitamin D concentrations and exposure time are necessary for its optimal effect in promoting decidualization. This provides important evidence for future research and clinical applications of vitamin D in treating infertility in women. Although the individual contributions of vitamin D to ESC decidualization are not fully understood, our future studies will focus on identifying related genes and analyzing how vitamin D regulates ESC decidualization through transcription factors. Our study could not detect E2 secretion from primary HESC using ELISA, likely due to levels being too low for this method. Future studies will use more sensitive techniques such as LC‐MS/MS to better understand E2 secretion in primary HESC during decidualization.

In our study, we discovered that vitamin D/VDR plays a crucial role in regulating the decidualization of human ESC through estrogen and estrogen receptor signaling pathways. Consequently, our findings provide a solid foundation for further analysis of how vitamin D deficiency affects endometrial receptivity. The vitamin D/VDR system has the potential to serve as a novel marker for the clinical assessment of endometrial receptivity and a promising therapeutic approach for infertility, recurrent implantation failure, or unexplained recurrent spontaneous abortions [9, 36–38]. Vitamin D is readily available, cost‐effective, and well‐tolerated by patients as a nutritional supplement, making it a promising option for clinical application. Future animal and clinical studies are necessary to confirm the effects of vitamin D supplementation on endometrial receptivity and determine optimal supplementation concentrations and strategies.

## 5. Conclusion

In conclusion, our study underscores the pivotal role of the vitamin D/VDR system in enhancing endometrial decidualization. In particular, we demonstrated that vitamin D/VDR significantly enhances the proliferation and decidualization of human ESC via estrogen signaling pathways. These findings suggest that vitamin D/VDR may serve as a promising biomarker and therapeutic target for improving endometrial receptivity and treating infertility.

## Author Contributions

Jing Guo: conceptualization, writing–original draft preparation, and funding acquisition. Xiangming Tian: investigation and visualization. Hailong Liu: conceptualization, review, and editing. Qun Lu: review and editing, supervision, and project administration. Guangming Cao: formal analysis, review and editing, supervision, project administration, and funding acquisition.

## Funding

The present study was financially supported by Beijing Natural Science Foundation (Project Nos. 7214234 and 7232069).

## Disclosure

All authors have reviewed and approved the final version of the manuscript for publication.

## Ethics Statement

The protocol was approved by the Institutional Review Board of Beijing Chao‐Yang Hospital (2024‐science‐791). Written informed consent was obtained from all participants.

## Conflicts of Interest

The authors declare no conflicts of interest.

## Supporting Information

Supporting information (Table S1): Detailed technical specifications of the commercial ELISA kits used to measure PRL and E2 concentrations in this study.

## Supporting information


**Supporting Information** Additional supporting information can be found online in the Supporting Information section.

## Data Availability

The data that support the findings of this study are available from the corresponding author upon reasonable request.
