# Supplementary material for: Unveiling the Role of Vitamin D/VDR in Promoting Endometrial Decidualization
Source: Int J Endocrinol. 2026 Feb 23;2026:1712178. doi: 10.1155/ije/1712178 (PMC12927962; doi:10.1155/ije/1712178)
Supplement: Supplementary file 1 — Supporting Information Additional supporting information can be found online in the Supporting Information section. [file IJE-2026-1712178-s001.docx]

**Table S1**. ELISA Kit Specifications for Measuring PRL and E2 Concentrations

| ELISA kit | Catalogue No. | Assay Format | Antibody Clones | Standard Range | LOD | CV (intra/inter) |
| --- | --- | --- | --- | --- | --- | --- |
| PRL | SEA846Hu | Sandwich | Capture: mouse mAb (clone N/S); Detection: biotin-polyclonal | 1.56–100μg/L | 0.65 μg/L | < 10 % / < 12 % |
| E2 | CEA461Ge | Competitive | Coated mAb (clone N/S); Biotin-E2 tracer | 12.35–1 000 ng/L | 4 ng/L | < 10 % / < 12 % |
